# The developmental biology of *Charnia* and the eumetazoan affinity of the Ediacaran rangeomorphs

**DOI:** 10.1126/sciadv.abe0291

**Published:** 2021-07-23

**Authors:** Frances S. Dunn, Alexander G. Liu, Dmitriy V. Grazhdankin, Philip Vixseboxse, Joseph Flannery-Sutherland, Emily Green, Simon Harris, Philip R. Wilby, Philip C. J. Donoghue

**Affiliations:** 1 Oxford University Museum of Natural History, University of Oxford, Parks Road, Oxford OX1 3PW, UK.; 2 British Geological Survey, Nicker Hill, Keyworth, Nottingham NG12 5GG, UK.; 3 School of Earth Sciences, University of Bristol, Life Sciences Building, Tyndall Avenue, Bristol BS8 1TQ, UK.; 4 Department of Earth Sciences, University of Cambridge, Downing Street, Cambridge CB2 3EQ, UK.; 5 Trofimuk Institute of Petroleum Geology and Geophysics, Prospekt Akademika Koptyuga 3, Novosibirsk 630090, Russia.; 6 Novosibirsk State University, Pirogova Street 1, Novosibirsk 630090, Russia.; 7 School of Geography, Geology and the Environment, University of Leicester, University Road, Leicester LE1 7RH, UK.

## Abstract

Molecular timescales estimate that early animal lineages diverged tens of millions of years before their earliest unequivocal fossil evidence. The Ediacaran macrobiota (~574 to 538 million years ago) are largely eschewed from this debate, primarily due to their extreme phylogenetic uncertainty, but remain germane. We characterize the development of *Charnia masoni* and establish the affinity of rangeomorphs, among the oldest and most enigmatic components of the Ediacaran macrobiota. We provide the first direct evidence for the internal interconnected nature of rangeomorphs and show that *Charnia* was constructed of repeated branches that derived successively from pre-existing branches. We find homology and rationalize morphogenesis between disparate rangeomorph taxa, before producing a phylogenetic analysis, resolving *Charnia* as a stem-eumetazoan and expanding the anatomical disparity of that group to include a long-extinct bodyplan. These data bring competing records of early animal evolution into closer agreement, reformulating our understanding of the evolutionary emergence of animal bodyplans.

## Teaser

Rangeomorphs represent a long-extinct group of eumetazoans with a bodyplan unlike anything alive today.

## INTRODUCTION

Divergences between the early metazoan lineages are estimated by molecular clock analyses to have occurred tens of millions of years before the earliest unequivocal fossil records of their crown groups (*1*). This mismatch is typically rationalized as a consequence of either systematic biases in the rock and fossil records, or inaccuracies in molecular clock methods (*2*, *3*). There is an emerging consensus that certain members of the Ediacaran macrobiota [~574 to 538 million years (Ma)], an infamously enigmatic group of fossilized macroscopic organisms whose affinities have long been contested, were early animals (*4*, *5*). Unfortunately, this general view is unsubstantiated for most taxa, and uncertainty over their phylogenetic affinities [e.g., (*6*)] means that these fossils have not contributed materially to debates surrounding metazoan divergence estimates. In large part, this uncertainty is a consequence of their unusual bodyplans, no better exemplified than by the frondose rangeomorphs. These are among the earliest components of the Ediacaran macrobiota (*7*) and, therefore, the oldest candidate metazoans among this assemblage. Description of rangeomorph anatomy is built largely upon specimens preserved in a two-dimensional (2D) cast-and-mold style [(*8*) though see (*9*, *10*)], leaving our understanding of their internal anatomy (and thus functional biology) unresolved [though see (*10*)], with competing hypotheses concerning even the most basic constructional parameters (e.g., the presence or absence of a stalk in different taxa) (*11*). All rangeomorphs have multiple orders of branching architecture (*12*, *13*), but the number of preserved orders is known to vary between taxa (*14*), and homology between orders across the group remains unclear. Patterns of morphogenesis also remain untested in communities of taxa, with published hypotheses (*15*–*17*) derived from either isolated single characters or simulated data.

Here, we attempt to leverage greater insight into the biology and phylogenetic affinity of Rangeomorpha (and thereby early animal evolution) by analyzing community assemblages that preserve a range of individuals of the iconic rangeomorph *Charnia masoni* at different sizes, interpreted as reflecting different developmental stages. We move beyond previous qualitative studies to quantitative analysis of populations to characterize morphogenesis of the rangeomorph bodyplan. We supplement this with x-ray tomographic microscopy (XTM) and computed tomography to establish the internal anatomical structure of rare three-dimensionally preserved specimens from the White Sea region of Russia. Our analyses reveal a highly connected and compartmentalized internal architecture, exhibiting branch origination points that are topologically constrained. These data do not support models that suggest substantial plasticity in rangeomorph growth programs [cf. (*17*)]. We exploit this new understanding of the biology of *Charnia* to constrain the phylogenetic affinity of rangeomorphs to stem-Eumetazoa, confirming a diverse Ediacaran history for this fundamental metazoan clade, and demonstrating the capacity for members of the Ediacaran macrobiota to inform the timing and patterns of character acquisition in early animal evolution.

### History of research into the morphogenesis of *Charnia*

Antcliffe and Brasier (*15*) observed that the smallest branches in *Charnia* were present at the apex of individual specimens and deduced that this was the position of the generative zone (singular), incompatible with a pennatulacean affinity. However, their interpretation is based solely on the relative size of apical branches and, in isolation, neither demonstrates the position of a generative zone (which requires comparison between specimens representing different developmental stages) nor precludes the presence of more than one generative zone. Hoyal Cuthill and Conway Morris (*16*) concluded that rangeomorphs had a simple morphogenetic pattern whereby branching structures grew isometrically, without discrete anatomical differences between taxa. However, this hypothesis was neither based in, nor tested using, populations of empirical data, comparisons among which might support or reject this model of morphogenesis. Wilby *et al.* (*18*) recognized that larger specimens of *Charnia* had fewer branches than might be expected, by counting the largest branching order, but preferred an ecological explanation for this phenomenon, and implied that different size cohorts have different developmental signatures. However, testing such a distinction would require investigation of other related characters, including the construction and growth of the largest branches, which that study does not offer. Butterfield (*10*) offered a conceptual model of the functional biology of *Charnia* and other rangeomorphs to inform hypotheses of anatomy (and therefore development) and phylogenetic affinity. Butterfield suggested that higher branching orders in *Charnia* (and presumably other rangeomorphs) reflect internal subdivisions, perhaps similar to cnidarian mesenteries, rather than the conventional interpretation as external surface features (*11*, *19*). However, the anatomical, developmental, and phylogenetic implications of this model remain untested.

## RESULTS

**Fig. 1:**
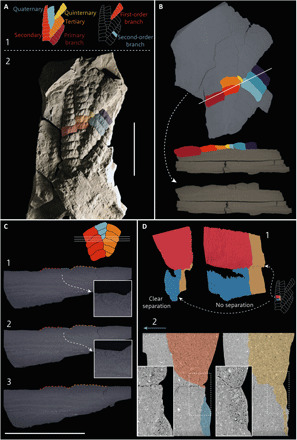
Three-dimensional anatomy of *Charnia.* *Charnia masoni* from the Verkhovka Formation, White Sea, Russia. (**A**) GCF (Geochron Core Facilities, Institute of Petroleum Geology and Geophysics, Novosibirsk, Russia) 2079-100 and associated terminology. (A-1) Left-hand schematic showing the problem of many successive orders of branches, using hierarchical terminology informed by developmental pattern. Right-hand schematic explains first-order branches and second-order branches, the terminology preferred in this paper. (A-2) GCF 2079-100 where colored second-order branches in specimen correspond to those in (B). (**B**) GCF 2079-100. Second-order branches illustrating that there is no central axis between/connecting individual first-order branches. Colored branches correspond to each other and original orthoslice provided for comparison. (**C**) GCF 2079-101. The color of the dashed lines corresponds to that in the schematic, with insets further showing the emergence of a first-order branch (blue in the schematic). No internal stalk is discernible. (**D**) Synchrotron radiation X-ray tomographic microscopy scan data from specimen GCF 2079-105. (D-1) Rendered model of specimen, showing the examined margin between two second-order branches. Separate branches are colored red and blue, and an area of apparent connectivity is shown in gold. (D-2) The nature of the boundary between second-order branches shown in individual orthoslices, with insets of regions of interest highlighted in white boxes. Branches in orthoslices are colored according to (D-1) for orientation of slices within the model. The top surface of the specimen is indicated by the blue arrow and the orthoslices are oriented parallel to the left-hand model in (C-1). Scale bars, 5 cm.

Five specimens of *C. masoni* from the Verkhovka Formation of the Onega Peninsula, White Sea, Russia (fig. S1) (*20*) were subjected to x-ray and computed tomographic analysis to establish the relationship between individual branching orders and the presence or absence of internal anatomical structures (Fig. 1). The frond of *Charnia* is composed of multiple levels of hierarchical branching, and previous descriptive ontological schemes for rangeomorphs describe these as first-, second-, third-, and fourth-order branches, or primary, secondary, tertiary, and quaternary branches: terms that are applied to branches of the same scale across an individual frond (*11*, *12*, *19*). However, this terminology does not necessarily reflect the process by which branches differentiated as the organism developed.

**Fig. 2:**
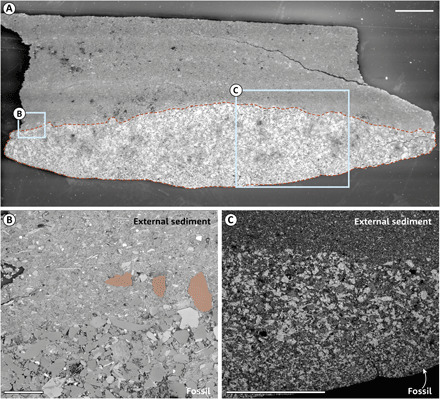
Preservation of three-dimensionally preserved specimens of *Charnia.* Charge contrast (A), back-scatter (B), and energy-dispersive x-ray (C) electron microscope images of specimen GCF 2079-105, from the Verkhovka Formation, White Sea, Russia. (**A**) A second-order branch in cross section (outlined by red broken line) and overlying sediment. Areas in (B) and (C) shown in light blue boxes. (**B**) Grains from the fossil cast are locally incorporated within the immediately overlying sediment (e.g., those highlighted). (**C**) Elemental map for Si, with lighter areas indicating greater abundance. A finer-grained fill defines the base of the cast. Scale bars, 1 mm (A and C) and 100 μm (B).

Our 3D reconstruction of anatomy resolves *Charnia* as exhibiting successive branching orders that are derived from each other (Fig. 1, A to C): The entire width of the frond is filled with branching units, with no evidence (or space) for an axial stalk from which branches may differentiate. Therefore, it follows that, in a developmental sense, *Charnia* exhibits tens of orders of branching (i.e., many “first”-order branches), themselves constructed of second- to fourth-order branches, an arrangement that (in terms of existing descriptive terminology) is incompatible with other rangeomorphs or with the anatomy of any other soft-bodied Ediacaran macroorganism (Fig. 1A-1). The interpretation of many tens of hierarchical branching orders is also incompatible with the contemporary hypotheses of homology between size-equivalent branching orders among other rangeomorphs [e.g., (*14*)]. Such an hypothesis offers no explanation for the asymmetric branching architecture exhibited by *Charnia*—where all branches are ultimately derived from a single branch, as opposed to emerging from a distinct central stalk. Nonetheless, our data suggest that the frond is composed of equally scaled, self-similar, modular units that we interpret as equivalent to the largest branching orders in other rangeomorphs, explaining the repetitive and architecturally limited branching pattern observed in *Charnia.* The largest of these repeated branching units in *Charnia* requires a descriptive term. We cannot use the classic term “frondlet”—“a centimeter scale module consist[ing] of inflated, self-similar branches” [(*12*), p. 1141]—because the anatomy of first-order branches in *Charnia* is not self-similar over three branching orders (*11*). In addition, a new term should not imply a growth mode. We therefore propose future terminological distinction between two existing descriptive schemes: first- to fourth-order, and primary to quaternary, branches. We propose that the primary to quaternary scheme should be used only when discussing developmental/morphogenetic aspects of frondose Ediacaran taxa. In this sense, in *Charnia*, there is ultimately only one primary branch from which all others are derived (Fig. 1A-1), and it is not currently clear that such a “primary branch” is present in all known rangeomorphs (e.g., those with a stalk). The term “first-order branch” has previously been used interchangeably with “primary” branch but carries fewer developmental connotations. We propose that, in the future, workers use the term first-order branch as a descriptive, anatomical term to define the fundamental unit from which the frond of *Charnia* is constructed (Fig. 1A-2), but do not, at this stage, invoke homology of this term with previously described first-order branches in other rangeomorphs.

Within a first-order branch, second-order branches [sensu (*19*)] are bound together medially; the boundary between the proximal portions of the second-order branches is indistinguishable, as opposed to the boundary between the distal portions (Fig. 1D). This implies that the medial portions of second-order branches were likely interconnected. Further support for this interpretation comes from the observation that three-dimensionally preserved specimens are entirely infilled with sediment, without evidence of partial three-dimensionality or partial collapse. Observed preservation is most compatible with the branches of the frond being interconnected.

SEM data from three-dimensionally preserved specimens indicate that there were likely multiple phases of sedimentary infill (Fig. 2); a layer of finer sediment is present at the base of the branch, whereas coarser sediment fills the remainder (Fig. 2C). These two infill phases are evident in closely associated third-order branches (Fig. 1D), implying that, at some point following death, the branches must have been “inflated” and presumably interconnected to allow sediment to circulate without baffling. A boundary appears to have existed between second-order branches at the time of infill [e.g., Dunn *et al*. (*11*), figure 10C], demonstrated by the ease with which individual second-order branches can be separated into discrete units with smooth faces. Tomographic data confirm these patterns (Fig. 1D). This interpretation is supported by the known anatomy of other rangeomorphs, e.g., *Hylaecullulus* (*24*) or *Fractofusus*, where there is no discrete common area from which isolated branches may diverge (as with the second-order branch of *Charnia*).

Third- and fourth-order branches have previously been shown to derive from the basal margin of second-order branches (*11*) on both sides of *Charnia* [assuming both faces of the organism are identical; (*11*, *18*)]. Our findings here suggest that each first-order branch differentiates between the third and sixth (from the base) second-order branch of the preceding first-order branch (table S1). This anatomical arrangement, in which *Charnia* does not have a stalk [defined as an axial structure running between branches, and distinct from a stem, which connects the holdfast to the frond (*11*)], distinguishes it from other rangeomorphs and increases the known branching permutations within the group, which now includes both monopodial (e.g., *Avalofractus*) and sympodial (*Charnia* here) arrangements. We find no evidence for structures lying internal to the second-order branch margin (Fig. 1, B to D).

**Fig. 3:**
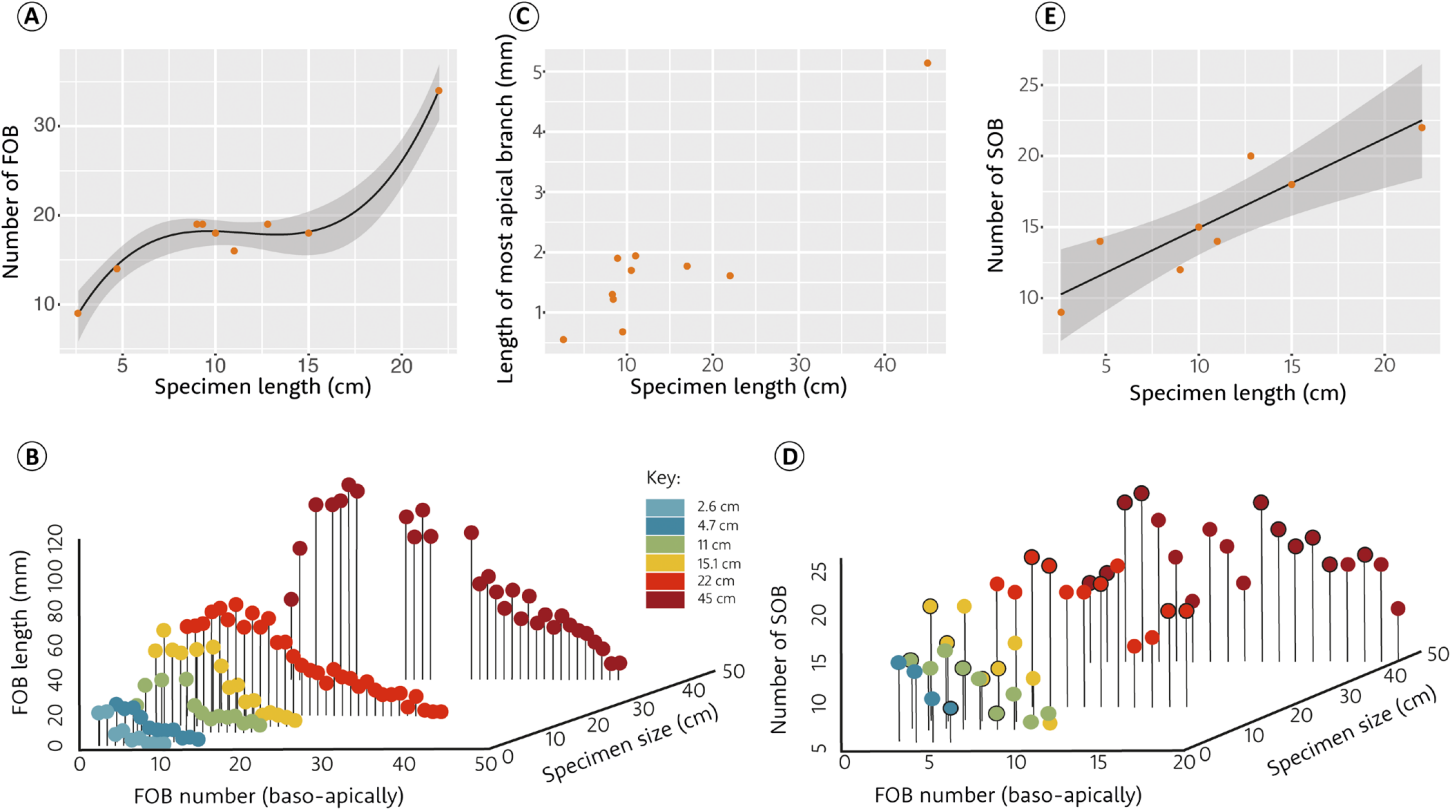
Morphogenesis in *Charnia.* The relationship between specimen length and the number of first- and second-order branches present. (**A**) Cubic regression between the total number of first-order branches and total specimen size (*P* = 0.00001) in unbroken black line, with confidence bars (conditional mean) in light gray. (**B**) Plot showing the relationship between specimen size, first-order branch number, and branch length for six *C. masoni* specimens from Charnwood Forest, Leicestershire. Individual specimens are marked by color [GSM (British Geological Museum) 105944, GSM 106084, GSM 105989, GSM 105997, LEIUG 2328, and GSM 105873]. (**C**) No significant relationship between the size of the most apical branch and total specimen length, omitting the largest specimen, which is an outlier. (**D**) Plot of specimen size, first-order branch number, and number of hosted second-order branches. Points with black outlines represent a minimum estimate for the number of second-order branches in cases where total numbers could not be confidently determined. Count includes one branch of each branch pair. (**E**) Linear relationship between maximal number of second-order branches (minimum estimate) per first-order branch and total specimen size (*P* = 0.003) in unbroken black line, with confidence bars (conditional mean) in light gray. FOB, first-order branches; SOB, second-order branches. Individual specimen plots can be found in fig. S3.

To characterize morphogenesis, we quantified the number of first-order branches and second-order branches across the frond in six specimens from Charnwood Forest, Leicestershire, UK, ranging from 2.7 to >45 cm in length (figs. S2 and S3). All specimens derive from the same bed [bed B (*21*)] and so reflect growth within similar paleoenvironmental conditions, which precludes major ecophenotypic causes of observed variation. Smaller specimens have fewer, smaller, first-order branches than larger specimens (Fig. 3, A and B), but the apical-most branches remain constant in size across most specimens, with no relationship between branch and specimen size as is observed across the rest of the frond (Fig. 3C). As these branches are size-equivalent in disparate specimens, the primary generative zone in *Charnia* is most likely to have been at the apical tip (*15*). However, the largest examined specimen exhibits larger apical branches than all other specimens (Fig. 3C). The apical-most first-order branches have fewer second-order branches than do first-order branches elsewhere within the frond, where the number of second-order branches remains approximately constant. The number of second-order branches on individual first-order branches increases with the overall size of the frond (Fig. 3, D and E). The relationship between specimen length and first-order branch length is best explained by logarithmic regression in the smallest specimens, but transitions via squared regression to linear in specimens that are ~10 cm or more in length, with the position of the longest branch moving basally down the frond in progressively larger specimens (fig. S3).

### Anatomical organization of *Charnia* and Rangeomorpha

The 3D preservation of the White Sea *Charnia* specimens permits consideration of their internal anatomy. Our results strongly support a model whereby second-order branches were interconnected. The sediment infill within *Charnia* is only possible if an opening exists within the specimen to allow sediment to enter, either a postmortem rupture of the external membrane or pre-existing (biological) openings within the organism. We do not see clear evidence for either of these within the preserved sections of the studied specimens. That an entire second-order branch and its derivative third-order branches demonstrate identical sediment fill (Fig. 2C) suggests that these branches were cast simultaneously. Therefore, if they were not originally interconnected, they would each have had to have individually burst, or been open to the environment, via an aperture portal of sufficient size to allow the largest grains to enter (107 μm), but we observe no evidence for either of these states. Therefore, we consider our data and the anatomy of *Charnia* to be consistent with a series of interconnected cavities.

The function of the interconnected compartments is unclear, but Butterfield (*10*) advanced a hypothesis that each individual second-order branch had its own gastrovascular cavity, with higher-order branches functioning as mesenteries. However, previous work has shown the independent mechanical flexibility of such structures [e.g., (*11*)], which is incompatible with a mesentery-like function. Furthermore, our XTM data show no evidence for internal strut-like projections, despite sufficient resolution in fossil preservation and x-ray tomography (Fig. 2C). While it remains possible that such structures could have decayed away post-mortem but before sediment infill, there is no evidence to support such a conclusion given the preservational fidelity of the organism’s exterior. Our taphonomic data do not preclude the presence of individual gastrovascular cavities in *Charnia* branches, but such an arrangement is difficult to reconcile with other rangeomorph taxa that display a much more elaborately branched anatomy [e.g., *Hylaecullulus* or *Avalofractus*]. An open cavity may be compatible with feeding via ciliary pumping, but further corroborative evidence [e.g., open pores] would be required to test this conjecture.

Our data reveal a highly compartmentalized internal anatomy for *Charnia*, reminiscent of the quilted pneu structure inferred by Seilacher (*22*) for what he perceived to be a clade of Vendozoa. Grazhdankin (*20*) presented evidence for an ellipsoidal cross-sectional profile for second-order branches in *Charnia*, but little direct evidence in support of this anatomical interpretation has been presented for other rangeomorphs. Our data corroborate Seilacher’s inference of a 3D modular structure, but the anatomy exhibits notable differences in detail. Seilacher envisaged the internal structure to be composed of struts that joined quiltings on opposing surfaces [see also Narbonne *et al*. (*23*)], effectively dividing the anatomy into discrete compartments and rendering Vendozoan anatomy without extant analog (*22*). Our data imply that while hierarchical branches are largely distinct from one another, they are connected via the point of branching in the largest two branching orders. Therefore, the branches are highly connected, as opposed to being fully divided. Thus, in the absence of a stalk in *Charnia*, the implication is that branches at all hierarchical levels (given that the smallest branching orders are derived from the second-order branch) are connected throughout the frond. This arrangement may not have been obtained for members of Rangeomorpha that had a stalk depending, of course, on the nature of stalk anatomy.

We document exterior-interior contiguous walls in *Charnia* (Fig. 1), which we consider to be flexible given previously published data suggesting that branches were able (rarely) to separate and twist, and third- and fourth-order branches were able to move independently of each other and their lower-order host branches [e.g., (*11*, *18*)], corroborating Seilacher’s conjectured flexible body wall with internal struts (*22*). Seilacher originally proposed a thin integument surrounding a chambered syncytium, whereas our data suggest an unmineralized and internally subdivided integument that had the capacity for growth and differentiation. We concur with Seilacher that this flexible support structure facilitated large body size among members of the Ediacaran macrobiota. Other authors have suggested the presence of internal struts in rangeomorph taxa [e.g., (*10*, *23*)], but our data indicate that the internal skeleton is continuous with (i.e., not differentiated from) the outer integument, with the discontinuous internal divisions marked by external sulci at the margins between branches (Fig. 2C).

### Morphogenesis of *Charnia*

**Fig. 4:**
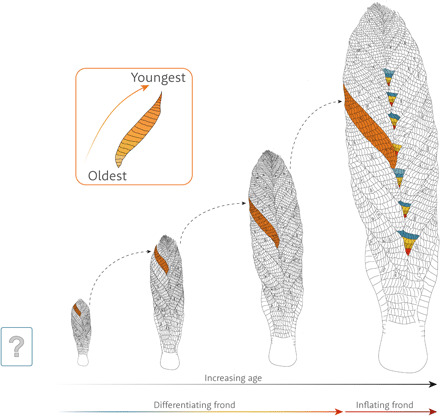
Model of morphogenesis in *C. masoni*. Green box represents an unknown stage in the life cycle. The shift in developmental mode (from primarily differentiation to primarily inflation) is illustrated, along with changes in relative branch measurements, such as the position of the longest branch (the longest branch in each specimen is shown in orange), which moves basally through development. A single first-order branch is traced through all illustrated growth permutations. Colored secondary branches in the largest *Charnia* illustrate the conserved number of second-order branches before the differentiation of another first-order branch. Inset: Presumed growth trajectory of second-order branches.

Together, these data allow us to infer a model of morphogenesis for *Charnia* (Fig. 4). Lateral branches (*11*) are described as a pair of first-order branches that derive from the margins of the holdfast disc rather than from the central axis of the frond. We currently cannot infer their growth relative to the rest of the main frond; however, their presence does not interfere with interpretation of the main frond and so they have not been considered within these analyses. The first-order branch at the base of the frond is the oldest (it contains the primary branch), and first-order branches differentiated in a baso-apical sequence, with new first-order branches added at the frond apex. The fundamental repeated unit, the first-order branch, appears to derive ultimately from a second-order branch of the preceding first-order branch (e.g., see the yellow and orange branches in Fig. 1B); its ultimate size is dictated by the position of the first-order branch that precedes it in sequence along the apical-basal axis. Because the number of second- to fourth-order branches within a first-order branch decreases along the main axis (along with first-order branch size) toward the apex, first-order branches must have grown through apical differentiation, as well as through inflation, for some time after they had moved from their apical position (Fig. 3 and fig. S3). Thus, differentiation continued at the apex of individual first-order branches across the (known) life cycle of a frond.

Smaller specimens of *Charnia* underwent relatively little inflation in the more apical branches (fig. S3, A and B), but differentiated branches relatively rapidly (Fig. 3A). The shift in specimen outline model fit from logarithmic to linear (fig. S3) indicates that more basal branches were becoming relatively larger in more mature specimens, as compared to smaller specimens, confirming that inflationary growth became more important as *Charnia* aged. Larger specimens of *Charnia* appear to have shown a greater increase in the rate of differentiation up to ~22 cm in length (Fig. 3A) but inflation of pre-existing branches kept pace. The largest specimen of *Charnia* shows larger apical branches than other specimens, indicating that it had ceased or slowed the differentiation of first-order branches from the apical generative zone (Fig. 3B).

These data suggest that *Charnia* exhibited different phases of growth and exhibited a shift in the primary developmental mode, from the differentiation of first-order branches to inflation of pre-existing first-order branches (Fig. 4). This shift was gradual and polarized along the principal frond axis, such that the outline shape of fronds is both regular and predictable, rather than exhibiting abrupt changes as might be anticipated by categorical shifts in growth mode. Among all of the specimens we have examined, none have exhibited any evidence of aberrant growth (Fig. 4 and fig. S2). Furthermore, if the patterns we describe in *Charnia* are general to Rangeomorpha, this would preclude morphogenetic models that interpret rangeomorph anatomy as highly mutable (*17*).

### Reconciling bodyplans among Rangeomorpha

**Fig. 5:**
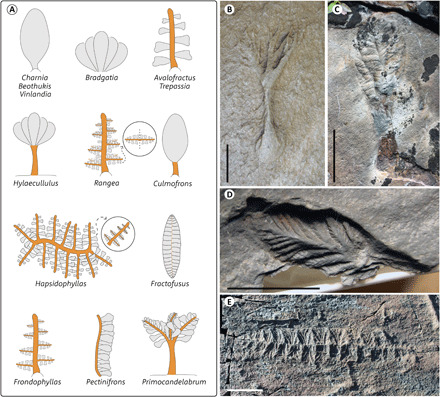
Homology scheme for Rangeomorpha. (**A**) Disparate rangeomorph bodyplans with potentially homologous branching orders shown in gray. Stalks and stems of unknown homology status are shown in orange. (**B** to **E**) Representative rangeomorph taxa on which this scheme is based, all images from Newfoundland, Canada. (B) *Primocandelabrum* sp., MUN Surface; (C) *Culmofrons plumosa*, MUN Surface; (D) *Avalofractus abaculus*, Spaniard’s Bay; (E) *Fractofusus misrai*, E Surface, Mistaken Point Ecological Reserve. Scale bars: B and D, 1 cm; C and E, 5 cm.

Our informed understanding of the anatomy and morphogenesis of *Charnia* provides a basis for testing established hypotheses of homology between rangeomorph bodyplans. Previously, a central stalk or similar structure has been identified as a fundamental aspect of all rangeomorph bodyplans (*14*, *16*). This assumption unites disparate rangeomorph anatomies, with the frond of *Charnia* interpreted as homologous to the frond of, for example, the genera *Avalofractus* or *Pectinifrons* (Fig. 5). However, our data demonstrate that *Charnia* does not have a stalk and, consequently, it is not possible to identify homology between bodyplans based on hierarchical branching patterns (Fig. 4). In *Charnia*, we observe first-order branches deriving from one another in sequence and, therefore, it is possible to find homology between the entire frond of *Charnia* and the single first-order branches in *Avalofractus* (which themselves do not appear to have a stalk). We identify a branching order that appears to exhibit a sympodial organization in all described rangeomorph taxa (Fig. 5A)—this is not the same branching order in every rangeomorph. This is the frame of reference from which it is most readily possible to rationalize disparate branching anatomies and distill the shared branching character of the group—the rangeomorph frondlet. We concur with previous assessments that all rangeomorphs have a branching unit comprising no fewer than three branching orders [e.g., (*13*)] (though they need not be identical in their fossilized expression, as is the case with *Charnia*) that are interconnected and sympodially organized. This is distinct from previous definitions of the frondlet, which are incompatible with our data because they are based on branching units with (at least) three orders of identical branching (*16*), an anatomy not seen in *Charnia*.

In *Charnia*, first-order branches differentiate from second-order branches, a pattern that bears notable similarity to the eccentric branching pattern described in the bush-like rangeomorphs *Hylaecullulus*, *Primocandelabrum* and *Bradgatia* (*24*). Eccentric branches exhibit the anatomy of the next highest branching order as an inferred response to in vivo damage. Eccentric branches are not present in all specimens, and their location across the frond is not consistent among an otherwise entirely consistent pattern of branching that reflects the fundamental morphogenetic pattern. This suggests that the mechanism allowing for the successive differentiation of first-order branches, which constitutes a consistent and coherent morphogenetic pattern in *Charnia*, was repressed in the bush-like rangeomorphs under normal growth conditions. This scheme of homology allows us to rationalize bodyplans with disparate branching patterns, providing a measure of confidence that observed anatomical variation in rangeomorphs can be explained by variation on a core pattern of branching morphogenesis.

### Phylogenetic affinity of *Charnia* and Rangeomorpha

*C. masoni* maintains differentiation of elements with concurrent axially delineated inflation, exhibits evidence for transitions in the primary developmental mode, and is compatible with indeterminate growth [the largest described specimens of *C. masoni* are >65 cm in length, reviewed in (*11*)], and the form of the organism is regular and predictable. This combination of characters is only otherwise seen within the Metazoa. Algae do not display a conserved form (*25*, *26*), and fungal fruiting bodies do not display the maintained differentiation of new elements (*27*, *28*) [reviewed in (*6*)]. Therefore, using these data in tandem with a large, multicellular organization, we conclude that there is no justification for considering an affinity for *Charnia* outside the animal total group.

Historically, attempts to resolve the affinity of Ediacaran macroorganisms, rangeomorphs, or *Charnia* in particular have been based on general comparisons to specific living groups (*29*) and/or arguments rooted in taphonomy (*22*) rather than homology. There has been a recent shift toward character-based phylogenetic analyses, but these have reached different conclusions for the phylogenetic position of *C. masoni* and/or Rangeomorpha among other Ediacaran taxa. Dececchi *et al*. (*30*) argued that the limited success in rationalizing Ediacaran taxa and extant groups results from characters of assumed phylogenetic utility potentially being convergent in origin. In their study, they therefore made no assumptions of trait history, effectively removing inference of homology. In contrast, the analysis of Hoyal Cuthill and Han (*31*) includes characters that are demonstrably nonhomologous (e.g., vertebrate sarcomeres, ctenophore ctenes, cnidarian septae, and annelid parapodia), providing no basis from which to recover a pattern of phylogenetic relationships. To better resolve the affinity of *C. masoni* within Opisthokonta, we compiled a phenotype dataset for living metazoans and nonmetazoan opisthokonts, upon which character states for *C. masoni* (based on the analyses herein) were scored. Despite the inevitably incomplete understanding of the biology of *Charnia*, we were able to score 80 of 178 characters (45%). Below, we justify the scoring of key characters for determining the phylogenetic position of *Charnia*.

#### Body tissues

These are present in all animals except Placozoa and Porifera (excluding homoscleromorphs, which have epithelia with basement membrane). *Charnia* displays body regionalization, minimally in the presence of a holdfast disc and a frond. Non-metazoans that have a similar grade of anatomical complexity to *Charnia* (e.g., kelp) are also known to have body tissues, which additionally justifies our scoring choice.

#### Anatomical polarity

This character requires multicellularity and therefore is contingent on that character. Anatomical polarity is defined as the ability to polarize the body along one or many axes. Anatomical polarity is present in all living animals and in *Charnia*.

#### Polarity type

This character does not differentiate between specific metazoan body axes (e.g., dorsal-ventral or oral-aboral), but orders successive body axes on the understanding that one must precede two must precede three. We conclude that *Charnia* has two principal body axes but lacks any evidence of a third left-right equivalent axis. We therefore score *Charnia* in the same way as the cnidarians and ctenophores, which also have two principal body axes.

#### First-order branches derive from one another

First-order branches—the highest branching order in a frondose bodyplan—derive directly from one another. This is an autapomorphy of *Charnia* and so all other multicellular taxa are scored as “absent.”

#### First-order branches comprise multiple other branching orders

First-order branches are constructed of at least three branching orders, which are internally interconnected. This is contingent on the presence of “first-order branches that derive from one another,” and so all groups absent for that character are scored as inapplicable.

#### Expanded surface area/volume ratio

An expanded surface area/volume ratio is well documented in rangeomorph taxa [e.g., (*32*)] based on the multiply branched architecture of the first-order branches. We do not consider this character to be present in any other lineages included in this matrix, and therefore, it represents an autapomorphy of *Charnia*.

#### Gastrovascular cavity

Gastrovascular cavities are known in cnidarians, ctenophores and bilaterians but are absent in placozoans (which use an external digestive sole) and sponges. We consider this character absent from *Charnia* because of the absence of any data to suggest either the presence of such a cavity or macroscopic openings through which food might be ingested, as well as the difficulties reconciling *Charnia*’s multiply branched anatomy with a vascular system. Some previous interpretations of *Charnia* (*10*) have advanced a colonial hypothesis for its body organization, which could imply the presence of many, smaller, oral openings. However, our growth characters do not imply a colonial mode of life, where one might expect to find branches able to grow independently of one another or exhibit variation in final form. Morphometric variation has previously been documented in *Charnia* (*11*) between different fossil localities, but within localities these parameters remain constrained. Such features would only then be compatible with a highly integrated colonial lifestyle, for example those found in modern sea pens, where there is a single, central, polyp, to which all others are connected, but this would require the original polyp to be a single second-order branch within the primary branch, which then exerted control over all other, successive branches. We view this as unlikely. Therefore, we consider the bodyplan of *Charnia* as incompatible with colonial metazoan comparators. For these reasons, we view our observation that a gastrovascular cavity is absent as the most likely scenario.

#### Coelenteron

A coelenteron is a gastrovascular cavity that is known in both cnidarians and ctenophores [reviewed in, e.g., (*33*)]. In the absence of any evidence for gastrovascular cavities in *Charnia*, given the absence of any macroscopic openings that could function as a mouth, we consider this character as absent.

#### Through-gut

Despite the presence of paired anal pores in ctenophores, we do not consider ctenophores as having a through-gut that is homologous with the bilaterian through-gut, following Zhao *et al*. (*34*). We do not observe any evidence for a gastrovascular cavity in *Charnia* with the absence of any macroscopic openings that could function as a mouth or anus, and so we consider this character absent.

#### Other characters

We score *Charnia* as unknown for equivocal characters, including the presence or absence of an aquiferous system with osculae. We use three characters to define the anatomy of *Charnia* with respect to other taxa in our matrix (first-order branches derive from each other, first-order branches comprise multiple other branching orders, and expanded surface area/volume ratio) but did not include additional characters derived from our morphogenetic analysis because in the absence of obvious points of homology between the rangeomorph bodyplan and those of living animals, any additional facets of anatomy would serve only to lengthen the branch leading to *Charnia* and not to recover the relationship between *Charnia* and living animals.

**Fig. 6:**
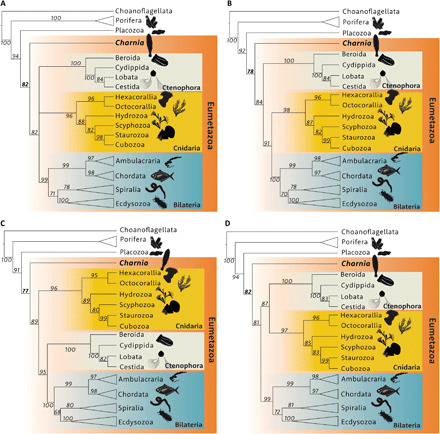
Phylogenetic analysis recovering *Charnia* as a stem-group eumetazoan. (**A**) Analysis without topological constraints recovers *Charnia* as a stem-group eumetazoan. (**B**) Analysis constraining monophyly of cnidarians and bilaterians recovers *Charnia* as a stem group to a clade encompassing cnidarians, bilaterians and ctenophores. (**C**) Analysis constraining monophyly of ctenophores and bilaterians recovers *Charnia* as a stem group to a clade encompassing cnidarians, bilaterians and ctenophores. (**D**) Analysis constraining monophyly of cnidarians and ctenophores recovers *Charnia* as a stem group to a clade encompassing cnidarians, bilaterians and ctenophores. Our analyses were run in Mr. Bayes 3.2.6 using a data matrix of 41 taxa and 182 characters. Posterior probabilities are shown for different nodes, and full trees can be found in fig. S4.

We subjected this dataset to Bayesian phylogenetic analysis, recovering a clade of eumetazoans (Cnidaria, Ctenophora and Bilateria), Placozoa lying outside Eumetazoa, and Porifera as the earliest-diverging metazoan lineage. We are not able to polarize the interrelationships of the three eumetazoan clades, but our phylogenetic scheme for extant lineages is largely uncontroversial [e.g., (*34*, *35*)], and we resolve *C. masoni* as a stem-eumetazoan with 82% support (Fig. 6A). To address the topological uncertainty surrounding the interrelationships of the Cnidaria, Ctenophora and Bilateria, we constrained the monophyly of three competing topologies: Coelenterata (a clade of cnidarians and ctenophores), Acrosomata (a clade of ctenophores and bilaterians), and a clade of cnidarians and bilaterians. *Charnia* is recovered as a sister to this clade—and so a stem-eumetazoan—with support 77% or greater in every case (Fig. 6, B to D).

## DISCUSSION

This is the first instance in which the affinity of a member of the Ediacaran macrobiota has been resolved among extant animals using a credible homology-based phylogenetic analysis. Resolution of a stem-eumetazoan affinity for *Charnia* corroborates some previous conjecture (*31*, *36*), but allows us to reject many previous interpretations of the organism’s phylogenetic affinity [e.g., (*22*, *37*, *38*)]. More germanely, the stem-eumetazoan affinity of *Charnia* has broad implications for understanding early animal evolution. Phylogenetic controversy concerning the interrelationships of nonbilaterian phyla has resulted in a number of potential scenarios for the evolution of key animal characters and, therefore, for the character states of potential ancestors. Key characters in the evolution of the nonbilaterian lineages include the acquisition of a gut and internal digestion, tissue and organ-grade anatomy with muscles, and nervous capacity. Many of these traits have different, complex, evolutionary histories under different phylogenetic scenarios. Notwithstanding these difficulties, we may still assess the anatomy of *Charnia* against that of living nonbilaterian lineages. Crown-placozoans feed via external digestion through a ventral sole [e.g., (*39*)], while cnidarians, ctenophores and bilaterians use an internal digestive cavity. Cnidarians, ctenophores and some bilaterians (e.g., xenacoelomorphs) have a blind gut, while the majority of living bilaterians display a through-gut with both a mouth and an anus. Ctenophores, cnidarians and bilaterians all have a nervous system; this is generally net-like in ctenophores and cnidarians, but there is some evidence for a nerve ring in *Nematostella* (*40*). Furthermore, recent work has shown the distinctiveness of the ctenophore nervous system, leading some to question the homology of animal nervous systems [reviewed in (*41*)]. Bilaterians often have a centralized nervous system, though there are notable (assumed derived) exceptions, for example, echinoderms. However, the homology of the centralized nervous system is not established [e.g., (*42*)]. Placozoans do not have a nervous system, but they do have fiber cells that are reactive against neuropeptides [e.g., (*39*)], confirming sensory capacity in this group. Cnidarians, ctenophores and bilaterians all have muscle cells, lacking in placozoans, but the type of cell varies with epithelial musculature dominating in cnidarians, and myocytes in bilaterians and ctenophores. Placozoans are not known to have differentiated tissues, or organs, while ctenophores, cnidarians, and bilaterians are. Crown-sponges feed through ciliary pumping and have a contractile pinacoderm. They are not known to have nervous capacity or a muscular system but may have precursors to these traits [e.g., (*43*)]. Homoscleromorphs are unique among sponges in having an epithelium, conferring tissue-grade anatomy. However, there is no evidence for tissue differentiation or organs, and no current phylogenies recover demosponges as the sister of all other poriferans, perhaps suggesting that their acquisition of epithelium is convergent [e.g., (*35*)].

There are reports that rangeomorphs may have been able to modulate branching architecture in vivo to respond to local environmental conditions. The bush-like rangeomorph *Primocandelabrum* displays branching architecture that may be density dependent: Furled first-order branches are significantly more common in less densely populated areas of seafloor (*44*). Similar flexibility in branching architecture, though notably only in third- and fourth-order branches across individual second-order branches, has been recognized in *Charnia* (*11*). These data do not preclude different taphonomic histories or differential local survival of furled versus unfurled specimens resulting in observed differences. Nevertheless, they may suggest that rangeomorphs were able to modulate some aspects of their branching anatomy, perhaps through a muscular system, though we consider this unlikely at present because there is no direct evidence for musculature or directed movement in rangeomorph fossils, and no evidence for retraction upon injury [e.g., figure 1F of Dunn *et al.* (*11*)]. Perhaps instead, *Charnia* was able to use a contractile epithelium, as mediated by the pinacoderm in sponges (*45*) or fiber cells in Placozoa.

Previous functional hypotheses have focused on the increased surface area that the frond-like anatomy of rangeomorphs confers and have forwarded an osmotrophic hypothesis of feeding (*32*). Butterfield (*10*), however, argues that such a hypothesis is unlikely because osmotrophy becomes increasingly unsustainable at increased body size, with attendant fluid dynamic effects. In this case, the observed anatomy of a series of hollow interconnected compartments could be compatible with other feeding hypotheses including, for example, ciliary pumping. No openings have been described in *Charnia* or other rangeomorphs, though it remains possible that *Charnia* had surface apertures that are beyond the limits of preservation, or in positions that are not typically preserved by cast-and-mold style preservation. Depending, ultimately, on the interrelationships of the nonbilaterian lineages, these data may suggest that the absence of a gut in crown-placozoans represents a primary absence.

We conclude that *Charnia* was a stem-eumetazoan, with potential evidence for sensory and contractile capacity, but with no compelling case for either muscles or a nervous system. This implies that *Charnia*, and other rangeomorphs, diverged from the eumetazoan lineage before the emergence of either of those key eumetazoan traits. A stem-eumetazoan affinity for *Charnia* suggests that a constrained and predictable anatomy—both across ontogeny and within populations—preceded the acquisition of muscles and a nervous system; presumably developing a fixed anatomy would be beneficial in the evolution of specialized organs. Despite a growing body of work suggesting that precursors of many key eumetazoan systems are present in crown-group sponges, which lack such a constrained anatomy, they have not converged on an organ-grade anatomy in over half a billion years of independent evolution.

Our interpretation of *Charnia* extends the minimum age of the eumetazoan total group to ~35 Ma before the Cambrian Period. Rangeomorphs appear in the rock record at ~574 Ma (*7*); therefore, phylogenetic bracketing requires that earlier diverging animal lineages and the metazoan LCA must have diverged earlier. Our data extend the minimum calibration on the animal crown node by some ~25 Ma (*1*) and confirm that the total-group Eumetazoa is minimally mid-Ediacaran in age, narrowing the gap between the fossil record and molecular clocks. The addition of Rangeomorpha to Eumetazoa substantially expands the disparity of eumetazoan bodyplans to include at least one that is entirely extinct and could not have been predicted from living eumetazoans. Our results indicate a richer pattern to early animal evolution than has been perceived hitherto, one in which the stem representatives of fundamental clades, like Eumetazoa, are not merely a subset of the characters exhibited by their living membership. Last, our analysis of *Charnia* establishes a framework in which the phylogenetic affinities of other members of the Ediacaran macrobiota may be constrained and, consequently, their evolutionary significance realized, further enriching our understanding of assembly of animal bodyplans, both extant and extinct.

## METHODS

### Fossil material

Fossil materials are deposited at the Geochron Core Facilities (GCF), Institute of Petroleum Geology and Geophysics, Novosibirsk, Russia, and the British Geological Survey (GSM). All specimens analyzed for individual growth analyses are shown in figs. S1 and S2.

### Tomographic analyses

Microfocus x-ray tomography was conducted at the University of Bristol, using a Nikon XT H 225 ST instrument with a Tungsten target with a 0.5-mm-thick copper filter, a current of between 147 and 156 μA, and a voltage of 215 kV. The ensuing data were reconstructed using Nikon VG Studios software. Synchrotron radiation x-ray microtomography was conducted at the X02DA TOMCAT beamline of the Swiss Light Source, Paul Scherrer Institute, Villigen, Switzerland (*46*). Specimens were measured using a LuAg:Ce 100-μm or LuAg:Ce 20-μm scintillator and a 4× objective lens (yielding reconstructed tomographic data with 1.625-μm voxel dimensions), at energy levels of 25 to 30 keV and exposure times of 250 to 700 ms. A total of 1501 projections were obtained equiangularly through 180° of rotation within the beam. Projections were postprocessed and rearranged into flat- and dark-field–corrected sonograms; reconstruction was performed on a 60-core Linux PC farm, implementing an optimized routine based on the Fourier transform method and a regridding procedure (*47*). Slice data were analyzed and manipulated using Avizo 9.4 (FEI Visualization Sciences Group). These specimens are interpreted not to have been subject to substantial tectonic deformation (*9*) and are not found in association with independent strain indicators, and so cannot be retrodeformed.

### Growth analyses

For quantitative analyses of growth, we assume the following:

1) Branches could differentiate during growth; we investigate first and second branching orders in this regard.

2) Branches could become larger but could not reduce in size once formed: Some variation in branch architecture (and resultant branch size) has been described as ecophenotypic [e.g., (*11*, *44*)], so only branch orders that showed a stable branch architecture arrangement between and across populations of *C. masoni* were assessed quantitatively. There is no available evidence to suggest that rangeomorphs were able to actively modulate branch size (e.g., hydrostatically during life). Recent data suggest that some rangeomorphs could alter morphology from concealed to displayed at certain branch orders (*44*), but such variation in branching anatomy has not been recognized in *Charnia* at quantified branching orders.

3) Total organism length varied only due to growth during life. Inflation or deflation of the holdfast theoretically remains a possibility, but there is no evidence to support such a suggestion for rangeomorphs to date.

4) All members of a single species follow a similar growth plan. We acknowledge that there may be morphometric ecophenotypic variation in *Charnia* (*11*), but we have attempted to compensate for this by quantitatively comparing only specimens derived from the same bedding surfaces.

### Model choice

The null model is of self-similar morphogenesis, with all elements growing at the same rate, because previous studies modeling rangeomorph growth have been conducted under these parameters (*16*). This would result in a direct, linear relationship between our variables (e.g., branch number and branch length). Therefore, we used regression analyses to test for a linear relationship between our variables against second- and third-order polynomial and logarithmic regressions. Residuals were checked using qqplots and model fit was assessed using the Akaike information criterion, with correction for small sample sizes to mitigate the likelihood of model overfitting.

Following previous work (*11*, *18*), we find that specimens of *C. masoni* from Charnwood Forest all exhibit a similar orientation on the bedding plane and, therefore, although they may have undergone tectonic deformation, all specimens from a single bedding plane will have been subjected to similar levels of deformation of the same morphological features. As such, we did not retrodeform these specimens.

### Phylogenetic analyses

Results were generated with majority consensus of 75,000 trees and run in MrBayes 3.2.6. We implemented the Mk model with a gamma distribution of modeled rate variation. The variable coding correction was applied as our matrix includes autapomorphies. Analyses were run for 10,000,000 generations, sampling every 100 generations. Effective sample size was larger than 200 and the deviation of split frequencies was less than 0.01. These data, in tandem with the use of Tracer 1.6, were used to assess convergence. Further details and additional references can be found in data file S2. Partial topological constraints were specified using the MrBayes commands “constraint” and “prset topologypr.”

### Electron microscopy

Electron microscopy (BSE) was carried out at the British Geological Survey, Keyworth on an FEI Company Quanta 600 environmental scanning electron microscope equipped with an Oxford Instruments INCA Energy 450 energy-dispersive x-ray microanalysis system (EDX) with a 500-mm^2^ Peltier-cooled (liquid nitrogen free) silicon drift x-ray detector in low vacuum mode. Two second-order branches (specimen GCF 2017-105) from the White Sea of Russia were embedded in Epotek 301 resin and polished using polycrystalline diamond paste to 1 μm. Microscopy was performed on uncoated samples in low vacuum mode using an accelerating voltage of 15 kV and either a working distance of 10 mm and a current of 0.28 nA (back-scatter and EDX) or 54.2 mm and 4.1 nA (charge contrast).

## SUPPLEMENTARY MATERIALS

Supplementary material for this article is available at http://advances.sciencemag.org/cgi/content/full/7/30/eabe0291/DC1

## Appendix

View/request a protocol for this paper from *Bio-protocol*.
